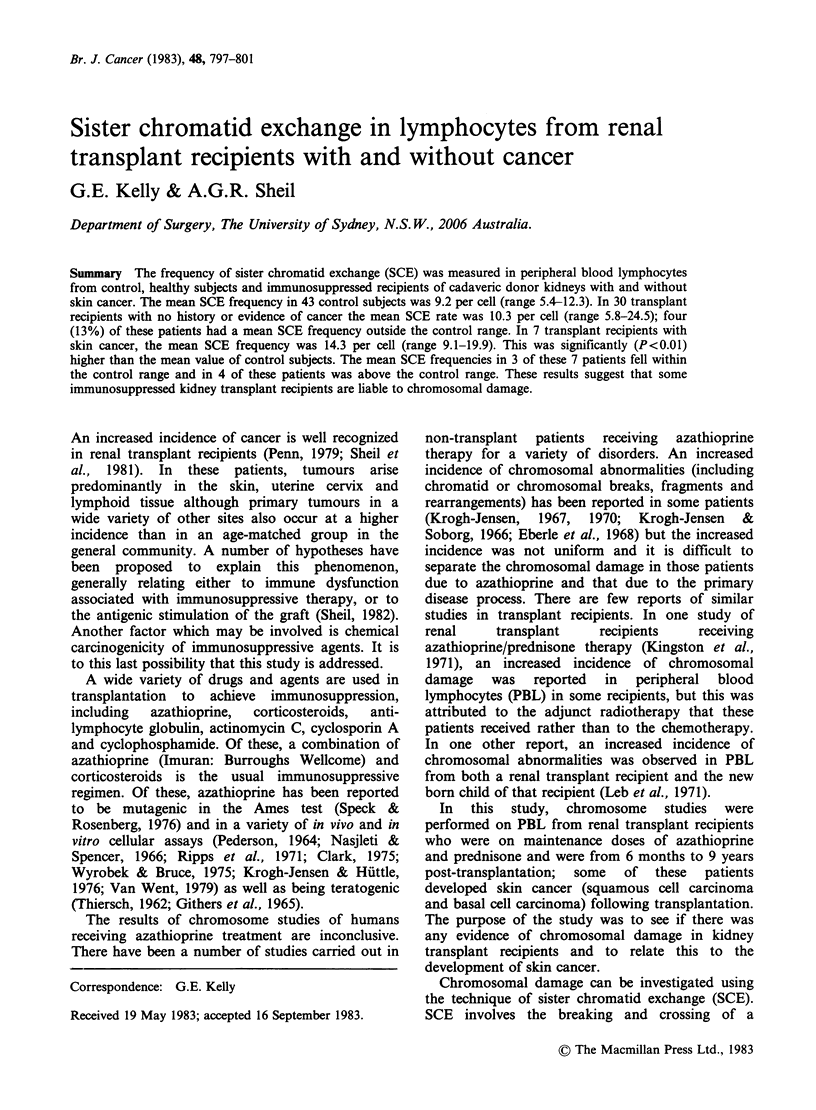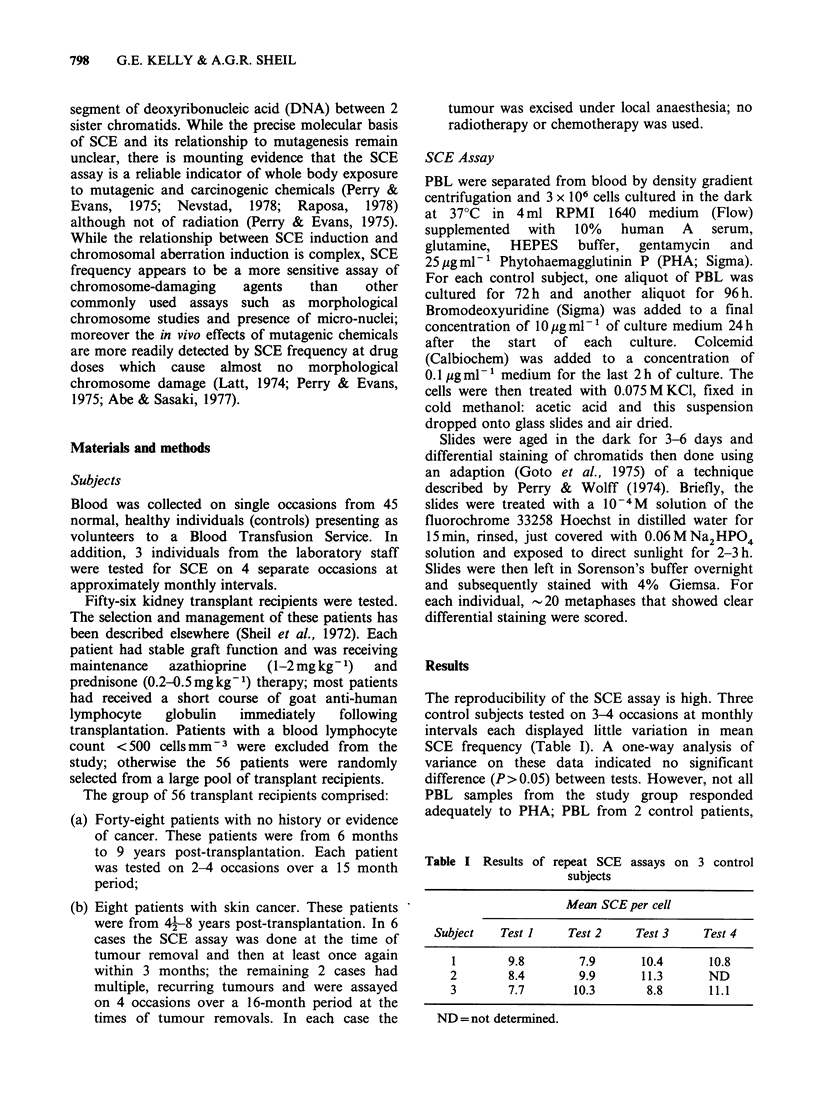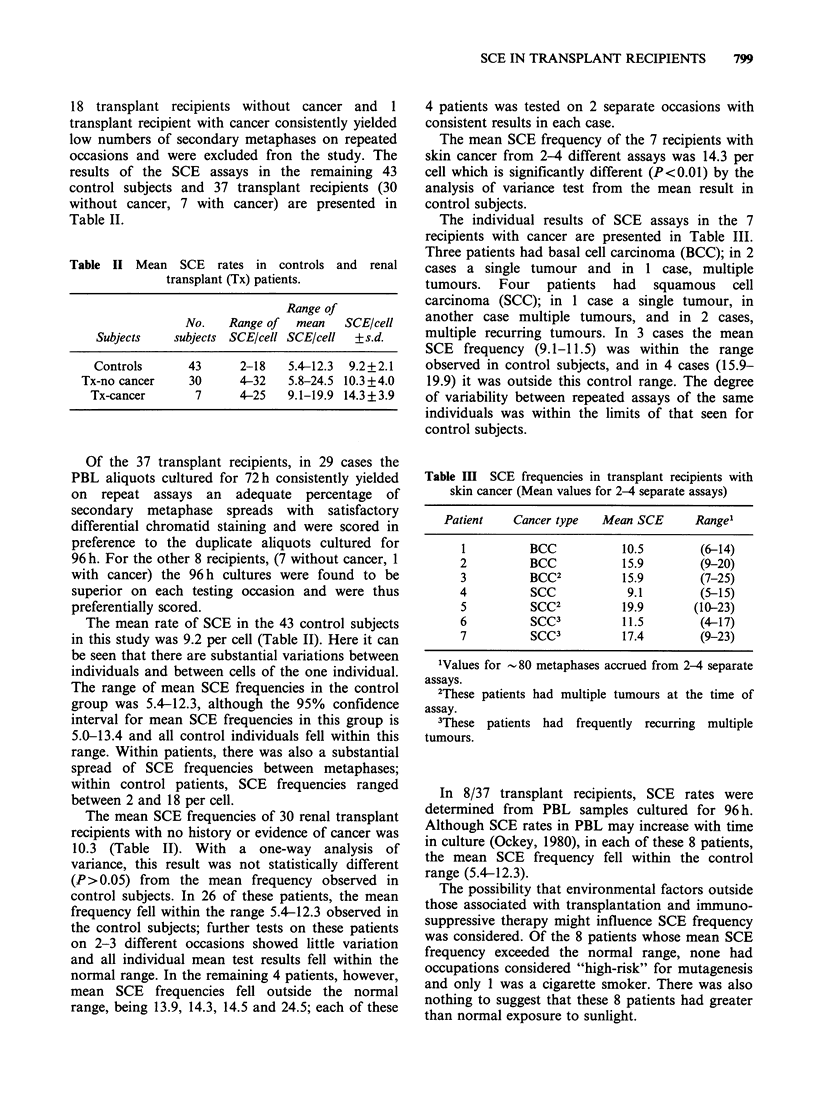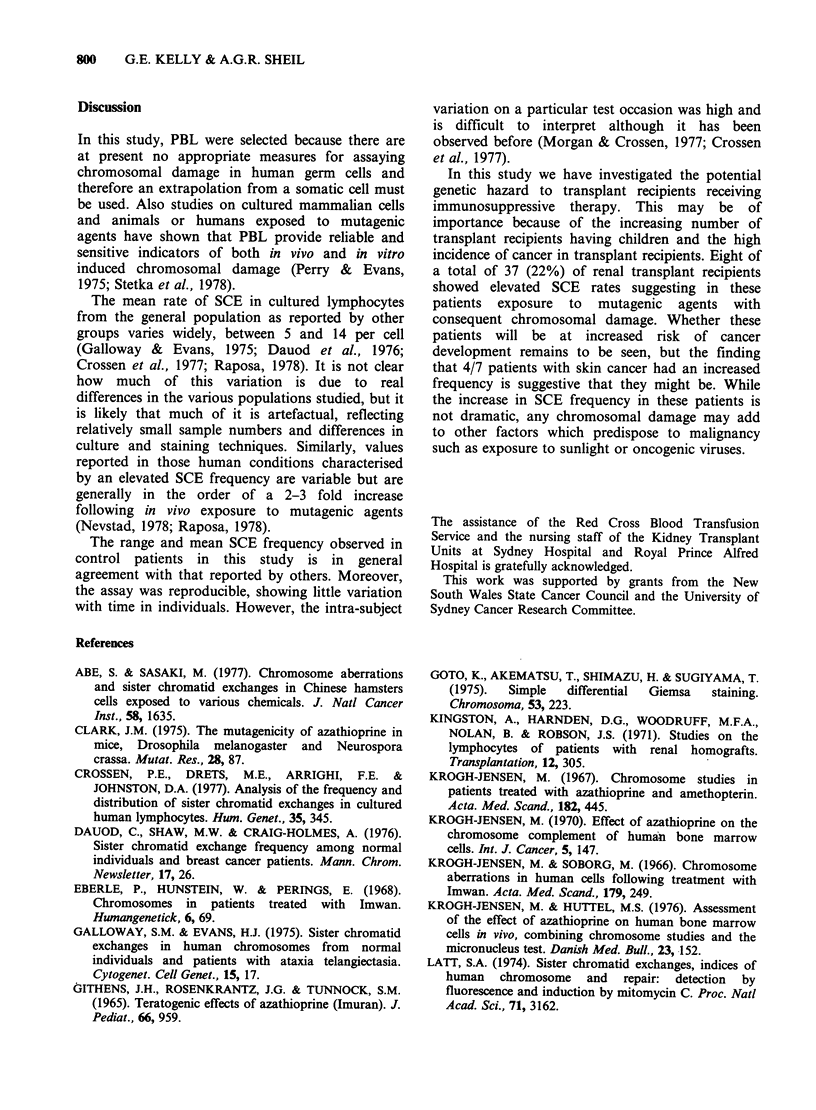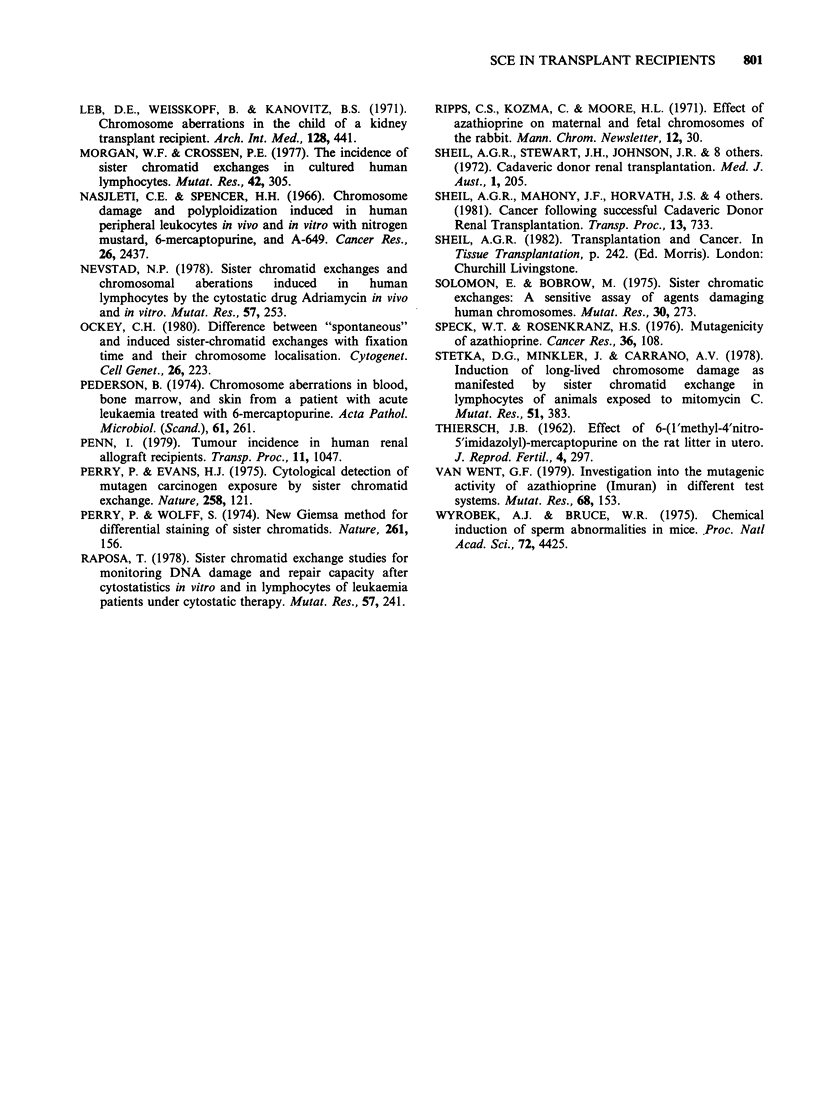# Sister chromatid exchange in lymphocytes from renal transplant recipients with and without cancer.

**DOI:** 10.1038/bjc.1983.269

**Published:** 1983-12

**Authors:** G. E. Kelly, A. G. Sheil

## Abstract

The frequency of sister chromatid exchange (SCE) was measured in peripheral blood lymphocytes from control, healthy subjects and immunosuppressed recipients of cadaveric donor kidneys with and without skin cancer. The mean SCE frequency in 43 control subjects was 9.2 per cell (range 5.4-12.3). In 30 transplant recipients with no history or evidence of cancer the mean SCE rate was 10.3 per cell (range 5.8-24.5); four (13%) of these patients had a mean SCE frequency outside the control range. In 7 transplant recipients with skin cancer, the mean SCE frequency was 14.3 per cell (range 9.1-19.9). This was significantly (P less than 0.01) higher than the mean value of control subjects. The mean SCE frequencies in 3 of these 7 patients fell within the control range and in 4 of these patients was above the control range. These results suggest that some immunosuppressed kidney transplant recipients are liable to chromosomal damage.


*Br. J. Cancer* (1983), **48**, 797–801

### Sister chromatid exchange in lymphocytes from renal transplant recipients with and without cancer

G.E. Kelly & A.G.R. Sheil

*Department of Surgery, The University of Sydney, N.S.W., 2006 Australia.*

**Summary** The frequency of sister chromatid exchange (SCE) was measured in peripheral blood lymphocytes from control, healthy subjects and immunosuppressed recipients of cadaveric donor kidneys with and without skin cancer. The mean SCE frequency in 43 control subjects was 9.2 per cell (range 5.4–12.3). In 30 transplant recipients with no history or evidence of cancer the mean SCE rate was 10.3 per cell (range 5.8–24.5); four (13%) of these patients had a mean SCE frequency outside the control range. In 7 transplant recipients with skin cancer, the mean SCE frequency was 14.3 per cell (range 9.1–19.9). This was significantly ($P<0.01$) higher than the mean value of control subjects. The mean SCE frequencies in 3 of these 7 patients fell within the control range and in 4 of these patients was above the control range. These results suggest that some immunosuppressed kidney transplant recipients are liable to chromosomal damage.

An increased incidence of cancer is well recognized in renal transplant recipients (Penn, 1979; Sheil *et al.,* 1981). In these patients, tumours arise predominantly in the skin, uterine cervix and lymphoid tissue although primary tumours in a wide variety of other sites also occur at a higher incidence than in an age-matched group in the general community. A number of hypotheses have been proposed to explain this phenomenon, generally relating either to immune dysfunction associated with immunosuppressive therapy, or to the antigenic stimulation of the graft (Sheil, 1982). Another factor which may be involved is chemical carcinogenicity of immunosuppressive agents. It is to this last possibility that this study is addressed.

A wide variety of drugs and agents are used in transplantation to achieve immunosuppression, including azathioprine, corticosteroids, anti-lymphocyte globulin, actinomycin C, cyclosporin A and cyclophosphamide. Of these, a combination of azathioprine (Imuran: Burroughs Wellcome) and corticosteroids is the usual immunosuppressive regimen. Of these, azathioprine has been reported to be mutagenic in the Ames test (Speck & Rosenberg, 1976) and in a variety of *in vivo* and *in vitro* cellular assays (Pederson, 1964; Nasjleti & Spencer, 1966; Ripps *et al.,* 1971; Clark, 1975; Wyrobek & Bruce, 1975; Krogh-Jensen & Hüttle, 1976; Van Went, 1979) as well as being teratogenic (Thiersch, 1962; Githers *et al.,* 1965).

The results of chromosome studies of humans receiving azathioprine treatment are inconclusive. There have been a number of studies carried out in non-transplant patients receiving azathioprine therapy for a variety of disorders. An increased incidence of chromosomal abnormalities (including chromatid or chromosomal breaks, fragments and rearrangements) has been reported in some patients (Krogh-Jensen, 1967, 1970; Krogh-Jensen & Soborg, 1966; Eberle *et al.,* 1968) but the increased incidence was not uniform and it is difficult to separate the chromosomal damage in those patients due to azathioprine and that due to the primary disease process. There are few reports of similar studies in transplant recipients. In one study of renal transplant recipients receiving azathioprine/prednisone therapy (Kingston *et al.,* 1971), an increased incidence of chromosomal damage was reported in peripheral blood lymphocytes (PBL) in some recipients, but this was attributed to the adjunct radiotherapy that these patients received rather than to the chemotherapy. In one other report, an increased incidence of chromosomal abnormalities was observed in PBL from both a renal transplant recipient and the new born child of that recipient (Leb *et al.,* 1971).

In this study, chromosome studies were performed on PBL from renal transplant recipients who were on maintenance doses of azathioprine and prednisone and were from 6 months to 9 years post-transplantation; some of these patients developed skin cancer (squamous cell carcinoma and basal cell carcinoma) following transplantation. The purpose of the study was to see if there was any evidence of chromosomal damage in kidney transplant recipients and to relate this to the development of skin cancer.

Chromosomal damage can be investigated using the technique of sister chromatid exchange (SCE). SCE involves the breaking and crossing of a

Correspondence: G.E. Kelly

Received 19 May 1983; accepted 16 September 1983.

© The Macmillan Press Ltd., 1983

segment of deoxyribonucleic acid (DNA) between 2 sister chromatids. While the precise molecular basis of SCE and its relationship to mutagenesis remain unclear, there is mounting evidence that the SCE assay is a reliable indicator of whole body exposure to mutagenic and carcinogenic chemicals (Perry & Evans, 1975; Nevstad, 1978; Raposa, 1978) although not of radiation (Perry & Evans, 1975). While the relationship between SCE induction and chromosomal aberration induction is complex, SCE frequency appears to be a more sensitive assay of chromosome-damaging agents than other commonly used assays such as morphological chromosome studies and presence of micro-nuclei; moreover the *in vivo* effects of mutagenic chemicals are more readily detected by SCE frequency at drug doses which cause almost no morphological chromosome damage (Latt, 1974; Perry & Evans, 1975; Abe & Sasaki, 1977).

### Materials and methods

### *Subjects*

Blood was collected on single occasions from 45 normal, healthy individuals (controls) presenting as volunteers to a Blood Transfusion Service. In addition, 3 individuals from the laboratory staff were tested for SCE on 4 separate occasions at approximately monthly intervals.

Fifty-six kidney transplant recipients were tested. The selection and management of these patients has been described elsewhere (Sheil *et al.*, 1972). Each patient had stable graft function and was receiving maintenance azathioprine (1–2 mg $kg^{-1}$) and prednisone (0.2–0.5 mg $kg^{-1}$) therapy; most patients had received a short course of goat anti-human lymphocyte globulin immediately following transplantation. Patients with a blood lymphocyte count $<500$ cells $mm^{-3}$ were excluded from the study; otherwise the 56 patients were randomly selected from a large pool of transplant recipients.

The group of 56 transplant recipients comprised:

(a) Forty-eight patients with no history or evidence of cancer. These patients were from 6 months to 9 years post-transplantation. Each patient was tested on 2–4 occasions over a 15 month period;

(b) Eight patients with skin cancer. These patients were from $4\frac{1}{2}$–8 years post-transplantation. In 6 cases the SCE assay was done at the time of tumour removal and then at least once again within 3 months; the remaining 2 cases had multiple, recurring tumours and were assayed on 4 occasions over a 16-month period at the times of tumour removals. In each case the tumour was excised under local anaesthesia; no radiotherapy or chemotherapy was used.

### *SCE Assay*

PBL were separated from blood by density gradient centrifugation and $3\times10^6$ cells cultured in the dark at 37°C in 4 ml RPMI 1640 medium (Flow) supplemented with 10% human A serum, glutamine, HEPES buffer, gentamycin and 25 $\mu g\,ml^{-1}$ Phytohaemagglutinin P (PHA; Sigma). For each control subject, one aliquot of PBL was cultured for 72 h and another aliquot for 96 h. Bromodeoxyuridine (Sigma) was added to a final concentration of 10 $\mu g\,ml^{-1}$ of culture medium 24 h after the start of each culture. Colcemid (Calbiochem) was added to a concentration of 0.1 $\mu g\,ml^{-1}$ medium for the last 2 h of culture. The cells were then treated with 0.075 M KCl, fixed in cold methanol: acetic acid and this suspension dropped onto glass slides and air dried.

Slides were aged in the dark for 3–6 days and differential staining of chromatids then done using an adaption (Goto *et al.*, 1975) of a technique described by Perry & Wolff (1974). Briefly, the slides were treated with a $10^{-4}$ M solution of the fluorochrome 33258 Hoechst in distilled water for 15 min, rinsed, just covered with 0.06 M $Na_2HPO_4$ solution and exposed to direct sunlight for 2–3 h. Slides were then left in Sorenson's buffer overnight and subsequently stained with 4% Giemsa. For each individual, ~20 metaphases that showed clear differential staining were scored.

### Results

The reproducibility of the SCE assay is high. Three control subjects tested on 3–4 occasions at monthly intervals each displayed little variation in mean SCE frequency (Table I). A one-way analysis of variance on these data indicated no significant difference ($P>0.05$) between tests. However, not all PBL samples from the study group responded adequately to PHA; PBL from 2 control patients,

**Table I** Results of repeat SCE assays on 3 control subjects

| | *Mean SCE per cell* | | | |
|---|---|---|---|---|
| *Subject* | *Test 1* | *Test 2* | *Test 3* | *Test 4* |
| 1 | 9.8 | 7.9 | 10.4 | 10.8 |
| 2 | 8.4 | 9.9 | 11.3 | ND |
| 3 | 7.7 | 10.3 | 8.8 | 11.1 |

ND = not determined.

18 transplant recipients without cancer and 1 transplant recipient with cancer consistently yielded low numbers of secondary metaphases on repeated occasions and were excluded fron the study. The results of the SCE assays in the remaining 43 control subjects and 37 transplant recipients (30 without cancer, 7 with cancer) are presented in Table II.

**Table II** Mean SCE rates in controls and renal transplant (Tx) patients.

| *Subjects* | *No. subjects* | *Range of SCE/cell* | *Range of mean SCE/cell* | *SCE/cell ±s.d.* |
|---|---|---|---|---|
| Controls | 43 | 2–18 | 5.4–12.3 | 9.2±2.1 |
| Tx-no cancer | 30 | 4–32 | 5.8–24.5 | 10.3±4.0 |
| Tx-cancer | 7 | 4–25 | 9.1–19.9 | 14.3±3.9 |

Of the 37 transplant recipients, in 29 cases the PBL aliquots cultured for 72 h consistently yielded on repeat assays an adequate percentage of secondary metaphase spreads with satisfactory differential chromatid staining and were scored in preference to the duplicate aliquots cultured for 96 h. For the other 8 recipients, (7 without cancer, 1 with cancer) the 96 h cultures were found to be superior on each testing occasion and were thus preferentially scored.

The mean rate of SCE in the 43 control subjects in this study was 9.2 per cell (Table II). Here it can be seen that there are substantial variations between individuals and between cells of the one individual. The range of mean SCE frequencies in the control group was 5.4–12.3, although the 95% confidence interval for mean SCE frequencies in this group is 5.0–13.4 and all control individuals fell within this range. Within patients, there was also a substantial spread of SCE frequencies between metaphases; within control patients, SCE frequencies ranged between 2 and 18 per cell.

The mean SCE frequencies of 30 renal transplant recipients with no history or evidence of cancer was 10.3 (Table II). With a one-way analysis of variance, this result was not statistically different ($P>0.05$) from the mean frequency observed in control subjects. In 26 of these patients, the mean frequency fell within the range 5.4–12.3 observed in the control subjects; further tests on these patients on 2–3 different occasions showed little variation and all individual mean test results fell within the normal range. In the remaining 4 patients, however, mean SCE frequencies fell outside the normal range, being 13.9, 14.3, 14.5 and 24.5; each of these 4 patients was tested on 2 separate occasions with consistent results in each case.

The mean SCE frequency of the 7 recipients with skin cancer from 2–4 different assays was 14.3 per cell which is significantly different ($P<0.01$) by the analysis of variance test from the mean result in control subjects.

The individual results of SCE assays in the 7 recipients with cancer are presented in Table III. Three patients had basal cell carcinoma (BCC); in 2 cases a single tumour and in 1 case, multiple tumours. Four patients had squamous cell carcinoma (SCC); in 1 case a single tumour, in another case multiple tumours, and in 2 cases, multiple recurring tumours. In 3 cases the mean SCE frequency (9.1–11.5) was within the range observed in control subjects, and in 4 cases (15.9–19.9) it was outside this control range. The degree of variability between repeated assays of the same individuals was within the limits of that seen for control subjects.

**Table III** SCE frequencies in transplant recipients with skin cancer (Mean values for 2–4 separate assays)

| *Patient* | *Cancer type* | *Mean SCE* | *Range*[1] |
|---|---|---|---|
| 1 | BCC | 10.5 | (6–14) |
| 2 | BCC | 15.9 | (9–20) |
| 3 | BCC[2] | 15.9 | (7–25) |
| 4 | SCC | 9.1 | (5–15) |
| 5 | SCC[2] | 19.9 | (10–23) |
| 6 | SCC[3] | 11.5 | (4–17) |
| 7 | SCC[3] | 17.4 | (9–23) |

[1]Values for ~80 metaphases accrued from 2–4 separate assays.
[2]These patients had multiple tumours at the time of assay.
[3]These patients had frequently recurring multiple tumours.

In 8/37 transplant recipients, SCE rates were determined from PBL samples cultured for 96 h. Although SCE rates in PBL may increase with time in culture (Ockey, 1980), in each of these 8 patients, the mean SCE frequency fell within the control range (5.4–12.3).

The possibility that environmental factors outside those associated with transplantation and immunosuppressive therapy might influence SCE frequency was considered. Of the 8 patients whose mean SCE frequency exceeded the normal range, none had occupations considered "high-risk" for mutagenesis and only 1 was a cigarette smoker. There was also nothing to suggest that these 8 patients had greater than normal exposure to sunlight.

### Discussion

In this study, PBL were selected because there are at present no appropriate measures for assaying chromosomal damage in human germ cells and therefore an extrapolation from a somatic cell must be used. Also studies on cultured mammalian cells and animals or humans exposed to mutagenic agents have shown that PBL provide reliable and sensitive indicators of both *in vivo* and *in vitro* induced chromosomal damage (Perry & Evans, 1975; Stetka *et al.*, 1978).

The mean rate of SCE in cultured lymphocytes from the general population as reported by other groups varies widely, between 5 and 14 per cell (Galloway & Evans, 1975; Dauod *et al.*, 1976; Crossen *et al.*, 1977; Raposa, 1978). It is not clear how much of this variation is due to real differences in the various populations studied, but it is likely that much of it is artefactual, reflecting relatively small sample numbers and differences in culture and staining techniques. Similarly, values reported in those human conditions characterised by an elevated SCE frequency are variable but are generally in the order of a 2–3 fold increase following *in vivo* exposure to mutagenic agents (Nevstad, 1978; Raposa, 1978).

The range and mean SCE frequency observed in control patients in this study is in general agreement with that reported by others. Moreover, the assay was reproducible, showing little variation with time in individuals. However, the intra-subject variation on a particular test occasion was high and is difficult to interpret although it has been observed before (Morgan & Crossen, 1977; Crossen *et al.*, 1977).

In this study we have investigated the potential genetic hazard to transplant recipients receiving immunosuppressive therapy. This may be of importance because of the increasing number of transplant recipients having children and the high incidence of cancer in transplant recipients. Eight of a total of 37 (22%) of renal transplant recipients showed elevated SCE rates suggesting in these patients exposure to mutagenic agents with consequent chromosomal damage. Whether these patients will be at increased risk of cancer development remains to be seen, but the finding that 4/7 patients with skin cancer had an increased frequency is suggestive that they might be. While the increase in SCE frequency in these patients is not dramatic, any chromosomal damage may add to other factors which predispose to malignancy such as exposure to sunlight or oncogenic viruses.

The assistance of the Red Cross Blood Transfusion Service and the nursing staff of the Kidney Transplant Units at Sydney Hospital and Royal Prince Alfred Hospital is gratefully acknowledged.

This work was supported by grants from the New South Wales State Cancer Council and the University of Sydney Cancer Research Committee.

### References

ABE, S. & SASAKI, M. (1977). Chromosome aberrations and sister chromatid exchanges in Chinese hamsters cells exposed to various chemicals. *J. Natl Cancer Inst.*, **58**, 1635.

CLARK, J.M. (1975). The mutagenicity of azathioprine in mice, Drosophila melanogaster and Neurospora crassa. *Mutat. Res.*, **28**, 87.

CROSSEN, P.E., DRETS, M.E., ARRIGHI, F.E. & JOHNSTON, D.A. (1977). Analysis of the frequency and distribution of sister chromatid exchanges in cultured human lymphocytes. *Hum. Genet.*, **35**, 345.

DAUOD, C., SHAW, M.W. & CRAIG-HOLMES, A. (1976). Sister chromatid exchange frequency among normal individuals and breast cancer patients. *Mann. Chrom. Newsletter*, **17**, 26.

EBERLE, P., HUNSTEIN, W. & PERINGS, E. (1968). Chromosomes in patients treated with Imwan. *Humangenetick*, **6**, 69.

GALLOWAY, S.M. & EVANS, H.J. (1975). Sister chromatid exchanges in human chromosomes from normal individuals and patients with ataxia telangiectasia. *Cytogenet. Cell Genet.*, **15**, 17.

GITHENS, J.H., ROSENKRANTZ, J.G. & TUNNOCK, S.M. (1965). Teratogenic effects of azathioprine (Imuran). *J. Pediat.*, **66**, 959.

GOTO, K., AKEMATSU, T., SHIMAZU, H. & SUGIYAMA, T. (1975). Simple differential Giemsa staining. *Chromosoma*, **53**, 223.

KINGSTON, A., HARNDEN, D.G., WOODRUFF, M.F.A., NOLAN, B. & ROBSON, J.S. (1971). Studies on the lymphocytes of patients with renal homografts. *Transplantation*, **12**, 305.

KROGH-JENSEN, M. (1967). Chromosome studies in patients treated with azathioprine and amethopterin. *Acta. Med. Scand.*, **182**, 445.

KROGH-JENSEN, M. (1970). Effect of azathioprine on the chromosome complement of human bone marrow cells. *Int. J. Cancer*, **5**, 147.

KROGH-JENSEN, M. & SOBORG, M. (1966). Chromosome aberrations in human cells following treatment with Imwan. *Acta. Med. Scand.*, **179**, 249.

KROGH-JENSEN, M. & HUTTEL, M.S. (1976). Assessment of the effect of azathioprine on human bone marrow cells *in vivo*, combining chromosome studies and the micronucleus test. *Danish Med. Bull.*, **23**, 152.

LATT, S.A. (1974). Sister chromatid exchanges, indices of human chromosome and repair: detection by fluorescence and induction by mitomycin C. *Proc. Natl Acad. Sci.*, **71**, 3162.

LEB, D.E., WEISSKOPF, B. & KANOVITZ, B.S. (1971). Chromosome aberrations in the child of a kidney transplant recipient. *Arch. Int. Med.*, **128**, 441.

MORGAN, W.F. & CROSSEN, P.E. (1977). The incidence of sister chromatid exchanges in cultured human lymphocytes. *Mutat. Res.*, **42**, 305.

NASJLETI, C.E. & SPENCER, H.H. (1966). Chromosome damage and polyploidization induced in human peripheral leukocytes *in vivo* and *in vitro* with nitrogen mustard, 6-mercaptopurine, and A-649. *Cancer Res.*, **26**, 2437.

NEVSTAD, N.P. (1978). Sister chromatid exchanges and chromosomal aberations induced in human lymphocytes by the cytostatic drug Adriamycin *in vivo* and *in vitro*. *Mutat. Res.*, **57**, 253.

OCKEY, C.H. (1980). Difference between "spontaneous" and induced sister-chromatid exchanges with fixation time and their chromosome localisation. *Cytogenet. Cell Genet.*, **26**, 223.

PEDERSON, B. (1974). Chromosome aberrations in blood, bone marrow, and skin from a patient with acute leukaemia treated with 6-mercaptopurine. *Acta Pathol. Microbiol. (Scand.)*, **61**, 261.

PENN, I. (1979). Tumour incidence in human renal allograft recipients. *Transp. Proc.*, **11**, 1047.

PERRY, P. & EVANS, H.J. (1975). Cytological detection of mutagen carcinogen exposure by sister chromatid exchange. *Nature*, **258**, 121.

PERRY, P. & WOLFF, S. (1974). New Giemsa method for differential staining of sister chromatids. *Nature*, **261**, 156.

RAPOSA, T. (1978). Sister chromatid exchange studies for monitoring DNA damage and repair capacity after cytostatistics *in vitro* and in lymphocytes of leukaemia patients under cytostatic therapy. *Mutat. Res.*, **57**, 241.

RIPPS, C.S., KOZMA, C. & MOORE, H.L. (1971). Effect of azathioprine on maternal and fetal chromosomes of the rabbit. *Mann. Chrom. Newsletter*, **12**, 30.

SHEIL, A.G.R., STEWART, J.H., JOHNSON, J.R. & 8 others. (1972). Cadaveric donor renal transplantation. *Med. J. Aust.*, **1**, 205.

SHEIL, A.G.R., MAHONY, J.F., HORVATH, J.S. & 4 others. (1981). Cancer following successful Cadaveric Donor Renal Transplantation. *Transp. Proc.*, **13**, 733.

SHEIL, A.G.R. (1982). Transplantation and Cancer. In *Tissue Transplantation*, p. 242. (Ed. Morris). London: Churchill Livingstone.

SOLOMON, E. & BOBROW, M. (1975). Sister chromatic exchanges: A sensitive assay of agents damaging human chromosomes. *Mutat. Res.*, **30**, 273.

SPECK, W.T. & ROSENKRANZ, H.S. (1976). Mutagenicity of azathioprine. *Cancer Res.*, **36**, 108.

STETKA, D.G., MINKLER, J. & CARRANO, A.V. (1978). Induction of long-lived chromosome damage as manifested by sister chromatid exchange in lymphocytes of animals exposed to mitomycin C. *Mutat. Res.*, **51**, 383.

THIERSCH, J.B. (1962). Effect of 6-(1′methyl-4′nitro-5′imidazolyl)-mercaptopurine on the rat litter in utero. *J. Reprod. Fertil.*, **4**, 297.

VAN WENT, G.F. (1979). Investigation into the mutagenic activity of azathioprine (Imuran) in different test systems. *Mutat. Res.*, **68**, 153.

WYROBEK, A.J. & BRUCE, W.R. (1975). Chemical induction of sperm abnormalities in mice. *Proc. Natl Acad. Sci.*, **72**, 4425.